# 3S – Systematic, Systemic, and Systems Biology and Toxicology

**DOI:** 10.14573/altex.1804051

**Published:** 2018

**Authors:** Lena Smirnova, Nicole Kleinstreuer, Raffaella Corvi, Andre Levchenko, Suzanne C. Fitzpatrick, Thomas Hartung

**Affiliations:** 1 Johns Hopkins University, Bloomberg School of Public Health, Center for Alternatives to Animal Testing (CAAT), Baltimore, MD, USA; 2 NIH/NIEHS/DNTP/NICEATM, RTP, NC, USA; 3 European Commission, Joint Research Centre (JRC), EU Reference Laboratory for Alternatives to Animal Testing (EURL ECVAM), Ispra, (VA), Italy; 4 Yale Systems Biology Institute and Biomedical Engineering Department, Yale University, New Haven, CT, USA; 5 Food and Drug Administration (FDA), Center for Food Safety and Applied Nutrition, College Park, MD, USA; 6 CAAT-Europe, University of Konstanz, Konstanz, Germany

**Keywords:** evidence-based toxicology, systems biology, repeated-dose toxicity, carcinogenicity, DART

## Abstract

A biological system is more than the sum of its parts – it accomplishes many functions via synergy. Deconstructing the system down to the molecular mechanism level necessitates the complement of reconstructing functions on all levels, i.e., in our conceptualization of biology and its perturbations, our experimental models and computer modelling. Toxicology contains the somewhat arbitrary subclass “systemic toxicities”; however, there is no relevant toxic insult or general disease that is not systemic. At least inflammation and repair are involved that require coordinated signaling mechanisms across the organism. However, the more body components involved, the greater the challenge to recapitulate such toxicities using non-animal models. Here, the shortcomings of current systemic testing and the development of alternative approaches are summarized.

We argue that we need a systematic approach to integrating existing knowledge as exemplified by systematic reviews and other evidence-based approaches. Such knowledge can guide us in modelling these systems using bioengineering and virtual computer models, i.e., via systems biology or systems toxicology approaches. Experimental multi-organon-chip and microphysiological systems (MPS) provide a more physiological view of the organism, facilitating more comprehensive coverage of systemic toxicities, i.e., the perturbation on organism level, without using substitute organisms (animals). The next challenge is to establish disease models, i.e., micropathophysiological systems (MPPS), to expand their utility to encompass biomedicine. Combining computational and experimental systems approaches and the challenges of validating them are discussed. The suggested 3S approach promises to leverage 21^st^ century technology and systematic thinking to achieve a paradigm change in studying systemic effects.

“I cannot say whether things will get better if we change; what I can say is they must change if they are to get better.”

Georg Christoph Lichtenberg (1742–1799)

“Systems thinking is a discipline for seeing wholes. It is a framework for seeing interrelationships rather than things, for seeing ‘patterns of change’ rather than static ‘snapshots’.”

Peter M. Senge (1947-), MIT

## 1 Introduction

Systematic, systemic, and systems sound very much alike, but they represent three different approaches in the life sciences. We will argue here that synergy between them is necessary to achieve meaningful understanding in biomedicine. Biology stands for the unperturbed “physiological” behavior of our model systems. Toxicology is certainly one of the more applied sciences studying the perturbation of model systems (pathophysiology); ultimately, all experimental medical research links to this. Here, we focus primarily upon examples from toxicology, which is the authors’ primary area of expertise, and the restriction to this area in the title appears prudent.

*Systematic* is a term most commonly used in the context of systematic reviews, i.e., evidence-based approaches that aim for a comprehensive, objective and transparent use of information. Born in the clinical and health care sciences, these approaches have gained significant traction in toxicology^1^ but have not had major impacts on other pre-clinical and biological areas. We will argue that this represents an omission and an opportunity, as the respective tools for evidence evaluation (quality scoring, risk-of-bias analysis, etc.) and integration (meta-analysis, combination of information streams, etc.) are widely applicable across scientific disciplines. The resulting condensation of information and mapping of knowledge deficits as well as the cross-talk with quality assurance, Good Practices, and reporting standards, yield valuable lessons on how the systematic evaluation of available scientific knowledge can accelerate the organization of vast, and rapidly expanding, knowledge generation.

*Systemic* views are primarily organism-level views on problems (the big-picture view), the opposite of studying smaller and smaller elements of the machinery. However, it is also thinking in terms of functionalities. Cell culture is starting to embrace this with the advent of complex co-cultures with multiplexed endpoints (Kleinstreuer et al., 2014) and organotypic cultures reproducing organ architecture and functionalities (microphysiological systems, MPS) (Marx et al., 2016), now even moving to multi-organ models of a human-on-chip / body-on-chip (Skardal et al., 2016; Watson et al., 2017). The concomitant emerging availability of human stem cells that can be used to produce high-quality organoids further adds to this revolutionary change (Suter-Dick et al., 2015), as shown recently for the BrainSphere organoid model (Pamies et al., 2017a, 2018a). Functional thinking can also be applied to cellular biology when considering toxic impact, for example, repair, recovery and resilience (Smirnova et al., 2015). We are returning to seeing the forest, not just individual trees.

*Systems* biology and, more recently, toxicology (Hartung et al., 2012, 2017a) aim to study systems behavior: “*Systems biology begins to recognize the limitations of the reductionist approach to biology*” (Joyner and Pedersen, 2011). In its detailed definition (Ferrario et al., 2014), it is based on a comprehensive study of our knowledge on these systems, which is translated into computer models, allowing virtual experiments/ simulations that can be compared to experimental results. Systems approaches require sufficient biological and physiological detail about the relevant molecular pathways, associated cellular behaviors, and complex tissue-level interactions, as well as computational models that adequately represent biological complexity while offsetting mathematical complexity. Bernhard Ø. Palsson wrote in his book *Systems Biology: Constraint Based Reconstruction and Analysis*, “*The chemical interactions between many of these molecules are known, giving rise to genome-scale reconstructed biochemical reaction networks underlying cellular functions*”. So, to some extent, the systems toxicology approach is systematic and systemic in view, but it brings in the additional aspects of knowledge organization using dynamic models of physiology.

**Fig. 1:**
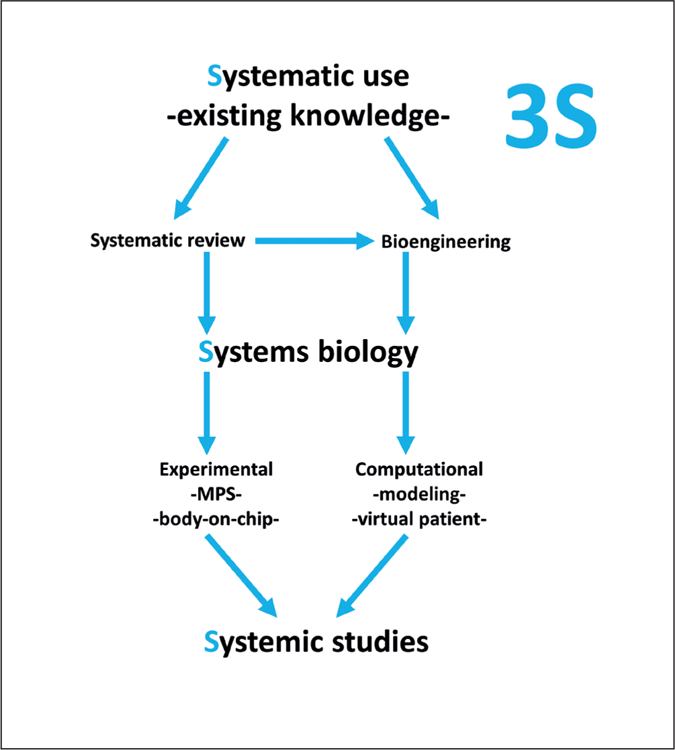
The 3S approach to study systemic phenomena

Figure 1 shows how these different components come together. This paper suggests that the traditional 3Rs approach, which has served us well to replace a substantial part of acute and topical toxicities, might need approaches along the 3S for systemic toxicity testing replacement. It suggests that systematic organization of existing knowledge be combined with experimental and computational systems approaches to model the complexity of (patho-)physiology.

## 2 Systematic biology and toxicology

Perhaps a better term would be “systematic review” of biology and toxicology. Similar to the term evidence, the concept of being systematic sounds like it must be a given for any scientific endeavor. Unfortunately, it is not. Most of us are drowning in a flood of information. The seemingly straightforward request of evidence-based medicine (EBM) to assess all available evidence quickly reaches limits of practicality. A systematic evaluation of the literature often returns (tens of) thousands of articles. Only very important questions, which must at the same time be very precise and very focused, warrant comprehensive efforts to analyze them. It is still worth the effort – as typically the result is strong evidence that is difficult to refute.

Earlier work in this area (Hoffmann and Hartung, 2006) led to the creation of the Evidence-based Toxicology Collaboration (EBTC) in 2011^1^. Developments have been documented (Griesinger et al., 2008; Stephens et al., 2013) and have gained acceptance (Aiassa et al., 2015; Stephens et al., 2016; Ågerstrand and Beronius, 2016). The field is developing very rapidly (Morgan et al., 2016; Mandrioli and Silbergeld, 2016). The fundamentals and advantages of evidence-based approaches were previously detailed in a dedicated article (Hartung, 2009a) that appeared earlier in this series (Hartung, 2017a), and are not repeated here. Noteworthy, the call for a systematic review of animal testing methods is getting louder and louder (Basketter et al., 2012; Leist et al., 2014; Pound and Brakken, 2014); the work of SYRCLE, the SY stematic Review Centre for Laboratory Animal Experimentation^2^ and CAMARADES^3^ (Collaborative Approach to Meta-Analysis and Review of Animal Data from Experimental Studies) is especially noteworthy. Here, we focus on two main points, one concerning opportunities for application in biology and other non-toxicology biomedical sciences, and the other framing the utility of systematic review in the context of systemic toxicities and systems toxicology.

Note that evidence-based approaches have a lot to offer also across diverse areas of biomedicine. Mapping what we know and what we do not, helps a field to focus research and resources, not only in areas where safety is at stake. In the clinical arena, EBM was the catalyst for many quality initiatives. Nobody wants to do research that is excluded from deriving authoritative conclusions by peers for quality (of reporting) reasons. A significant portion of irreproducible science could be avoided by using evidence-based approaches (Hartung, 2013; Freedman et al., 2015).

It should also be commented that in the context of systemic toxicities, we first of all need a systematic review of the traditional test methods, the information that they provide, and the decision contexts in which they are used. This was the unanimous recommendation of the roadmap exercise on how to overcome animal-based testing for systemic toxicology (Basketter et al., 2012; Leist et al., 2014). Systematic review was also suggested as a necessary element for a mechanistic validation (Hartung et al., 2013); this represents a key opportunity for the validation of both adverse outcome pathways (AOP) and mechanistic experimental models such as MPS. Lastly, systems toxicology should be based on a comprehensive analysis of biological systems characteristics, again calling for systematic literature analysis.

## 3 Systemic biology and toxicology

Systemic biology is not a common term – probably “physiology” covers it best, though much of physiology is studied in isolated organs. With the flourishing of molecular and cellular biology and biochemistry, systemic biology has been less prominent over the last few decades. However, the need to understand molecular and mechanistic findings in the context of an intact organism is obvious. This is one of the arguments for whole animal experimentation that are more difficult to refute. In fact, it is the use of genetically modified animals in academic research that is driving the steady increase of animal use statistics after three decades of decline (Daneshian et al., 2015).

Regulatory assessment of the complex endpoints of repeated-dose toxicity (RDT), carcinogenicity, and developmental and reproductive toxicity (DART), which are often grouped under systemic toxicities, still relies heavily on animal testing. Arguably, there is hardly any toxicity in nature that is not systemic as even topical effects such as skin sensitization include inflammatory components involving leukocyte infiltration and other acute effects, e.g., lethality, involve many parts of the organism. But, the aforementioned areas of toxicology represent the best examples of systemic toxicology, in which new approaches are needed but implementation is not straightforward.

The following section first addresses the limitations of current systemic toxicity testing and then reviews the alternative approaches that were developed in these areas of systemic toxicity in the last decades to waive testing or reduce the number of animals used.

The shortcomings of the current paradigm have been discussed earlier (Hartung, 2008a, 2013; Basketter et al., 2012; Paparella et al., 2013, 2017); some studies that cast doubt as to their performance are summarized in Table 1, using the more factual references, though the balance between opinion and evidence is difficult in the absence of systematic reviews (Hartung, 2017b). However, they stress the need for the strategic development of a new approach (Busquet and Hartung, 2017), especially for the systemic toxicities.

Alternative approaches range from the individual test methods (e.g., the cell transformation assay for carcinogenicity and the zebrafish and embryonic stem cell embryotoxicity tests for reproductive toxicity) to animal reduction approaches such as the International Council for Harmonisation of Technical Requirements for Pharmaceuticals for Human Use (ICH) strategy for carcinogenicity of pharmaceuticals and the extended-one-generation reproductive toxicity study. Currently, these areas are being revitalized owing to the broad recognition of the shortcomings of current *in vivo* testing requirements and the current regulatory environment (e.g., the European REACH and Cosmetic Regulation, the US amendment to the Toxic Substance Control Act (TSCA), i.e., the Lautenberg Chemical Safety for the 21^st^ Century Act). More recent developments aimed at a more human-relevant chemical assessment, which rely on the integration of different sources of information, are also described.

The assessment of repeated-dose systemic toxicity, carcinogenicity and developmental and reproductive toxicity represent essential components of the safety assessment of all types of substances, being among the endpoints of highest concern. As such, their assessment still relies mainly on animal tests. Progress toward replacing this paradigm is summarized in the following sections.

### 3.1 Repeated-dose systemic toxicity

Repeated chemical treatment, usually on a daily basis, from several days to life-long exposure, is key to the hazard assessment of substances as it covers toxicokinetic aspects, i.e., adsorption, distribution, metabolism and excretion (ADME), as well as toxicodynamics with the potential of all organ effects and interactions. The present testing schemes are based on rodent or non-rodent studies performed for 28 days (subacute toxicity), 90 days (subchronic toxicity), or 26–102 weeks (chronic toxicity). These tests typically form the basis for identifying hazards and their characterization, especially no-effect-levels (NOEL). This approach rests upon the key assumption that the animal models are representative of human ADME and effects. In fact, the enormous differences in ADME represented a key reason for drug attrition two decades ago (attrition has dropped from 40–60% to nowadays 10% (Kennedy, 1997; Kubinyi, 2003; Kola and Landis, 2004)), as the development of a portfolio of *in vitro* and *in silico* tools has drastically improved the situation as reviewed earlier in this series (Tsaioun et al., 2016). The toolbox is neither perfect nor complete but, as discussed in the context of developing a roadmap for improvment (Basketter et al., 2012; Leist et al., 2014), there was general consensus among the experts involved that the missing elements are feasible and in reach. For example, epithelial barrier models (Gordon et al., 2015) as input into physiology-based (pharmaco-/toxico-) kinetic (PBPK) modelling were identified as a key opportunity for modelling RDT and were recently the subject of the Contemporary Concepts in Toxicology 2018 meeting “Building a Better Epithelium”.

The Adler et al. (2011) report already compiled the many partial solutions to RDT. The problem is how to integrate these elements into a testing strategy that provides predictivity of human toxicity that is equivalent or greater than that of an animal study. This is a difficult question to answer, as in most cases we do not actually know how predictive the animal studies are due to the absence of human data. A notable exception is in the area of topical toxicities such as skin sensitization, where the predictive performance of the animal studies against human data has been shown to be essentially equivalent to the reproducibility of the animal data (Kleinstreuer et al., 2018).

We can start by asking how reproducible they are and how well different laboratory animal species predict each other. An important analysis conducted by Wang and Gray (2015) gives us an idea: very little. They compared earlier RDT findings with the non-cancer pathologies observed in cancer bioassays in rats and mice of both genders run by the National Toxicology Program for 37 substances. They concluded: “*Overall, there is considerable uncertainty in predicting the site of toxic lesions in different species exposed to the same chemical and from short-term to long-term tests of the same chemical*.” Although this study was done for only 37 chemicals, it gives us a hint that there is no reason to assume that the predictivity of rodent data for humans will be any better. For a larger scale comparison, the key obstacle is the lack of harmonized ontologies and reporting formats for RDT (Hardy et al., 2012a,b; Sanz et al., 2017). Very often it is unclear whether effects for certain organs or systemic effects were not reported because (a) they were assessed but not found and not reported as negative data; (b) there were already other organ toxicities at lower doses and, thus, the data on remaining organs was omitted or not assessed, or (c) only one organ was the focus of the study and the remaining and/or systemic effects were out of scope for the given study. Therefore, the standardized curation of databases with detailed organ effects is a resource-intensive problem, and there are currently none that facilitate widespread reproducibility assessments. Independent of the specific site of toxic manifestations, however, it is relatively easy to compare NOELs across studies. Using our machine-readable database from the REACH registration process (Luechtefeld et al., 2016a), such comparisons between 28- and 90-day studies showed strong discrepancies (Luechtefeld et al., 2016b). A systematic evaluation of RDT studies will enable further analysis of the current testing paradigm.

Given these problems, it will be very difficult to model such findings with a test strategy (Chen et al., 2014). Our t^4^ workshop on *Adversity in vitro* (report in preparation) in the context of the Human Toxome Project (Bouhifd et al., 2015), took a different approach: Based on the observation that the majority of chemicals are quite promiscuous, i.e., start perturbing the same cellular pathways in a relatively narrow concentration range, it appears feasible to define *in vitro* benchmark doses at which adversity starts using a set of complementary cell-based assays (Judson et al., 2016). Quantitative *in vitro* to *in vivo* extrapolation based on exposure data (plus some safety factors) should allow definition of exposures necessary to reach such tissue concentrations. Without necessitating a prediction of which organs would be affected, a safe use range would be established. In fact, the current risk assessment paradigm also makes little use of which organ exhibits toxic effects first but relies upon the most sensitive endpoint to define a benchmark / no-effect dose. Obviously, this does not work for substances whose molecular initiating events (MIE) are not reflected in the cell test battery to derive benchmark doses or NOELs. This means that over time this should be complemented with specific assays for those substances whose effects may be missed with this approach. Read-across strategies could add safety measures to such an approach, i.e., besides defining the safe dose, read-across and other *in silico* tools could provide alerts for where to add additional safety factors. In cases where human exposure is not sufficiently below doses that can reach critical tissue concentrations, it will be necessary to follow a more investigative toxicological approach, i.e., a mechanistic evaluation addressing the human relevance of the findings.

Biological models for different organs, e.g., liver, kidney, lung or brain, have been established and new culture techniques, especially in form of 3D organoids and MPS, are expected to solve present *in vitro* testing issues concerning long-term culturing, absence of relevant immune cells (Hengstler et al., 2012) and availability of fully mature cell phenotypes. Stem cells, especially induced pluripotent stem cells (iPSC), are a major source of tissue and cell models not available otherwise. Therefore, research on the generation of 2D cultures and 3D tissues from stem cells is of high importance. The formalization of our mechanistic knowledge via adverse outcome pathways (AOP) (Leist et al., 2017) further helps to assess whether these models are relevant. New prospects come from systems approaches, where human complexity is either modelled experimentally or virtually, as discussed below. The European Commission-funded Horizon 2020 consortium EU-ToxRisk was in fact set up to integrate advances in cell biology, omics technologies, systems biology and computational modelling to define the complex chains of events that link chemical exposure to toxic outcome in the areas of RDT, developmental and reproductive toxicity (Daneshian et al., 2016). The vision of EU-ToxRisk, which builds on the activities started by the SEURAT-1 EU framework project, is to progress towards an animal-free toxicological assessment based on human cell responses and a comprehensive mechanistic understanding of cause-consequence relationships of chemical adverse effects^4^.

### 3.2 Carcinogenicity

Substances are defined as carcinogenic if after inhalation, ingestion, dermal application or injection they induce (malignant) tumors, increase their incidence or malignancy, or shorten the time to tumor occurrence. It is generally accepted that carcinogenesis is a multi-hit/multi-step process from the transition of normal cells into cancer cells via a sequence of stages and complex biological interactions, strongly influenced by factors such as genetics, age, diet, environment and hormonal balance (Adler et al., 2011). Although attributing observed cancer rates to individual specific causes remains a challenge, the fraction of all cancers currently attributed to exposure to carcinogenic pollutants is estimated to range from less than 1% to 10–15% to as high as 19% (Kessler, 2014; Colditz and Wei, 2012; Anand et al., 2008; President’s Cancer Panel, 2010; GBD 2013 Risk Factors Collaborators, 2013).

For nearly half a century, the 2-year rodent cancer bioassay was widely considered the “gold standard” for determining the carcinogenic potential of a chemical and OECD Test Guidelines (TG) exist since 1981 (Madia et al., 2016). Its adequacy to predict cancer risk in humans, however, is the subject of considerable debate (Gottmann et al., 2001; Alden et al., 2011; Knight et al., 2006a,b; Paules et al., 2011) (Tab. 1). Recently, Paparella and colleagues (2017) conducted a systematic analysis of challenges and uncertainties associated with the cancer bioassay. Notably, extrapolating from rodents to humans and quantitative risk estimation has limited accuracy (Knight et al., 2006b; Paparella et al., 2017; Paules et al., 2011). Moreover, the rodent bioassay, as originally designed, does not take into account windows of susceptibility over the life-time, and so may not have adequate sensitivity to detect agents, such as endocrine active chemicals, that alter susceptibility to tumors (Birnbaum et al., 2003). Furthermore, these studies are very time- and resource consuming, taking up to three years to completion, and the high animal burden has raised ethical concerns.

From a regulatory perspective, the gradual recognition of non-genotoxic mechanisms of carcinogenesis (that do not involve direct alterations in DNA) has complicated the established relationship between genotoxicity and carcinogenicity and has challenged the conventional interpretation of rodent carcinogenicity results in terms of relevance to human cancer (Hengstler et al., 1999; Waters, 2016). Because of the default assumption in regulatory decision-making regarding the presumed linearity of the dose-response curve for genotoxic carcinogens, the classification of carcinogens as genotoxic or non-genotoxic became an essential but highly controversial component of cancer risk assessment.

The area of carcinogenicity has been very quiet for decades, but in recent years it has been revitalized due to broad recognition of the shortcomings of current regulatory *in vivo* testing requirements, and the awareness of information gaps in legislation that limit or ban the use of animals (e.g., European REACH Regulation (EC) No. 1907/2006 and Cosmetic Regulation (EC) No 1223/2009).

Table 2 shows some steps on the road to replacing the traditional paradigm, some of them are detailed in the following paragraphs.

#### Genotoxicity assays

Beginning in the late 1960s, highly predictive short-term genotoxicity assays were initially developed to screen for carcinogens. This led to a variety of well-established *in vitro* assays and, since the 1980s, to their respective OECD TGs that have been used successfully to predict genotoxicity, label chemical substances and inform cancer risk assessment. However, these tests are not at present considered to fully replace animal tests currently used to evaluate the safety of substances (Adler et al., 2011). In the last decade, several activities have been carried out worldwide with the aim of optimizing strategies for genotoxicity testing, both with respect to the basic *in vitro* testing battery and to *in vivo* follow-up tests. This was motivated by the scientific progress and significant experience of 40 years of regulatory toxicology testing in this area.

One of the major gaps identified was the need to ensure that *in vitro* tests do not generate a high number of false positive results, which trigger unnecessary *in vivo* follow-up studies, hence generating undesirable implications for animal welfare (Kirkland et al., 2005). The recommendations from a workshop organized by ECVAM (Kirkland et al., 2007) and from an EURL ECVAM strategy paper (EURL ECVAM, 2013a) on how to reduce genotoxicity testing in animals have contributed to several international initiatives aiming to improve the existing *in vitro* genotoxicity tests and strategy and to identify and evaluate new test systems with improved specificity, while maintaining appropriate sensitivity. The outcome of this work led to the revision of OECD TGs for genotoxicity.

Meanwhile, the *in vitro* micronucleus test, which was the first test to be evaluated by ECVAM through retrospective validation (Corvi et al., 2008), is acquiring an increasingly prominent role in the genotoxicity strategy. It has in fact been proposed as the assay to be used in a two-test battery together with the Ames test (Kirkland et al., 2011; Corvi and Madia, 2017). Further *in vitro* methods are being developed and validated, especially aiming at a full replacement, as in the case of genotoxicity assays in 3D human reconstructed skin models, and for a better understanding of modes of action (MoA) using toxicogenomics-based tests and biomarker assays (Corvi and Madia, 2017).

#### Transgenic mouse models

Transgenic mouse model tests are possible alternatives to the classical two-year cancer bioassay owing to their enhanced sensitivity as predictors of carcinogenic risk to humans (Tennant et al., 1999). In fact, these models have a reduced tumor latency period (6–9 months) to chemically-induced tumors and may result in a significant reduction in the use of experimental animals (20–25 animals/sex/treatment group) (Marx, 2003). A study coordinated by ILSI/HESI (ILSI HESI ACT, 2001; MacDonald et al., 2004) led to the initial acceptance by pharmaceutical regulatory agencies of three primary models: p53^+/−^, Tg.AC and rasH2 model, to be used *in lieu* of a second species full carcinogenicity bioassay (ICH, 2009).

#### Cell transformation assays

*In vitro* cell transformation assays (CTA) for the detection of potential carcinogens have been in use for about four decades. Transformation involves several events in the cascade potentially leading to carcinogenesis and is defined as the induction of phenotypic alterations in cultured cells that are characteristic of tumorigenic cells (LeBoeuf et al., 1999; Combes et al., 1999). Despite the long experience in the use of CTAs, the intense and prolonged activities at the OECD from 1997 to 2016, and the performance of ECVAM and JaCVAM validation studies (EURL ECVAM, 2012, 2013b), the assays were adopted as OECD Guidance Documents (OECD, 2015, 2016), but they have so far not been adopted as OECD TGs in their own right. Among the obstacles to the development of an OECD TG for the Syrian Hamster Embryo (SHE) Cell Transformation Assay (SHE CTA) was the lack of a coordinated full validation. The combination of a detailed review paper (DRP) and a prospective limited validation study triggered the need for additional analyses by the OECD expert group (OECD, 2007; Corvi et al., 2012). This also demonstrates that a DRP cannot be considered equivalent to a retrospective validation. Moreover, with the lack of an Integrated Approach to Testing and Assessment (IATA) or alternative testing strategy available for carcinogenicity and since there was common agreement that the assay should not be used as a stand-alone, no strategy was available on how to apply it for regulatory decision-making. This dilemma, “What comes first: the chicken or the egg, the test method or the testing strategy (or IATA)?” raises the question whether in the future the OECD should accept only new methods associated to a well-defined testing strategy or an IATA. A better characterization of the performance of the CTA to detect non-genotoxic carcinogens was considered important because the data collected in the DRP were biased towards genotoxic carcinogens, which reflects data available in the public domain. Another recurring concern was that the mechanistic basis of cell transformation and the link to tumorigenesis are not yet completely elucidated, which hampers interpretation of the findings from such an assay.

During the course of the OECD CTA activities, the regulatory framework in Europe changed considerably with the ban on animal testing for cosmetics (Hartung, 2008c) coming into force and the REACH evaluation of industrial chemicals commencing (Hartung, 2010b). This has put a huge burden on industry, which is limited in the use of *in vivo* tests to confirm results from *in vitro* tests, and on regulators, who have to assess carcinogenicity potential without *in vivo* data, leading to a more cautious uptake of *in vitro* tests to support assessment of such a critical endpoint. Many of these considerations apply also to the CTA based on Bhas 42 cells.

#### IATAs for non-genotoxic carcinogens

Non-genotoxic carcinogens contribute to an increased cancer risk by a variety of mechanisms that are not yet directly assessed by international regulatory approaches. In April 2014, the OECD WNT recognized that the CTA alone was insufficient to address non-genotoxic carcinogenicity and that a more comprehensive battery of tests addressing different non-genotoxic mechanisms of carcinogenicity would be needed in the future. This discussion led to the identification of the need for an IATA to properly address the issue of non-genotoxic carcinogenicity and where the CTA, together with other relevant assays, could fit. Under the auspices of the OECD, an expert working group was thus set up to examine the current international regulatory requirements and their limitations with respect to non-genotoxic carcinogenicity, and how an IATA could be developed to assist regulators in their assessment of non-genotoxic carcinogenicity (Jacobs et al., 2016). Moreover, the working group is tasked to review, describe and assess relevant *in vitro* assays with the aim of tentatively organizing them into levels of testing, following the adverse outcome pathway format such that possible structure(s) of the future IATA(s) can be created. Different *in vitro* methods are in fact already available as research tools to study a number of potential non-genotoxic mechanisms, such as oxidative stress or inhibition of gap junction intercellular communication (GJIC) (Basketter et al., 2012; Jacobs et al., 2016). Recent work has focused on mapping *in vitro* high-throughput screening (HTS) assays, e.g., from the ToxCast research program, to the hallmarks of cancer (Kleinstreuer et al., 2013a) and the characteristics of carcinogens (Chiu et al., 2018). However, these methods cannot currently be used to reliably predict carcinogenic potential; rather they are useful to better understand the mechanistic basis of effects elicited by a compound, as demonstrated by use in International Agency for Research on Cancer (IARC) monographs, within a weight of evidence strategy (i.e., IATA).

#### Toxicogenomics-based test methods for carcinogenicity

Toxicogenomics for the study of carcinogenicity has been applied to several *in vitro* and short-term *in vivo* test systems (Vaccari et al., 2015; Schaap et al., 2015; Worth et al., 2014). For example, the EU-Framework Project carcinoGENOMICS, which aimed at developing *in vitro* toxicogenomics tests to detect potential genotoxicants and carcinogens in liver, lung and kidney cells, also assessed the preliminary reproducibility of the assay using different bioinformatics approaches (Doktorova et al., 2014; Herwig et al., 2016). Potential applications of toxicogenomics-based assays are clarification of mode of action (MoA), hazard classification, derivation of points of departure (PoD) and prioritization (Paules et al., 2011; Waters, 2016). Among these, the targeted use of transcriptomics tests for MoA determination seems to be the preferred application. However, there is still limited implementation of transcriptomics in regulatory decision-making, as discussed in a recent workshop featuring multi-sector and international perspectives on current and potential applications of genomics in cancer risk assessment organized by the Health and Environmental Sciences Institute (HESI), Health Canada and Mc Gill University in Montreal in May 2017. Even though companies make use of transcriptomics-based tests to guide internal decisions, the uncertainty on how these data would be interpreted by regulators is among the main roadblocks identified for submission of data. In addition, lack of validation and regulatory guidance were considered roadblocks (Corvi et al., 2016).

#### Systematic approaches to carcinogenicity assessment

Identification and incorporation of important, novel scientific findings providing insights into cancer mechanisms is an increasingly essential aspect of carcinogen hazard identification and risk assessment. In recent years, the IARC realized that its process for classifying human carcinogens was complicated by the absence of a broadly accepted, systematic method to evaluate mechanistic data to support conclusions regarding human hazard from exposure to carcinogens. First, no broadly accepted systematic approach was in place for identifying, organizing, and summarizing mechanistic data for the purpose of decision-making in cancer hazard identification. Second, the agents documented and listed as human carcinogens showed a number of characteristics that are shared among many carcinogenic agents. Hence, ten key properties that human carcinogens commonly exhibit and that can encompass many types of mechanistic endpoints were identified. These characteristics were used to conduct a systematic literature search focused on relevant endpoints that provides the basis for an objective approach to identifying and organizing results from pertinent mechanistic studies (Smith et al., 2016).

An example of a comprehensive systematic literature review was recently compiled by Rodgers et al. (2018). Here epidemiologic studies published since 2007, which were related to chemicals previously identified as mammary gland toxicants, were reviewed. The aim was to assess whether study designs captured relevant exposures and disease features, including windows of susceptibility, suggested by toxicological and biological evidence of genotoxicity, endocrine disruption, tumor promotion, or disruption of mammary gland development. Overall, the study added to evidence of links between environmental chemicals and breast cancer.

Beside systematic reviews, IATA can be considered approaches that integrate and weight all relevant existing evidence in a systematic manner to guide the targeted generation of new data, where required, and to inform regulatory decision-making regarding potential hazard and/or risk (e.g., IATA for non-genotoxic carcinogens as described above).

#### Alternative approaches to rodent long-term carcinogenicity studies for pharmaceuticals

Mainly due to deficiencies of animal carcinogenicity studies and based on some extensive data reviews, representatives of the pharmaceutical industry have leveraged decades of experience to make a proposal for refining the criteria for when carcinogenicity testing may or may not be warranted for pharmaceuticals. In August 2013, an ICH Regulatory Notice Document (RND) was posted by the Drug Regulatory Authorities (DRAs) announcing the evaluation of an alternative approach to the two-year rat carcinogenicity test (ICH Status Report, 2016). This approach is based on the hypothesis that the integration of knowledge of pharmacological targets and pathways together with toxicological and other data can provide sufficient information to anticipate the outcome of a two-year rat carcinogenicity study and its potential value in predicting the risk of human carcinogenicity of a given pharmaceutical. The rationale behind this proposal was supported by a retrospective evaluation of several datasets from industry and drug regulatory agencies, which suggests that up to 40–50% of rat cancer studies could be avoided (ICH Status Report, 2016; Sistare et al., 2011; van der Laan et al., 2016).

A prospective evaluation study to confirm the above hypothesis is ongoing. The industry sponsors are encouraged to submit a carcinogenicity assessment document (CAD) to address the carcinogenic potential of an investigational pharmaceutical and predict the outcome and value of the planned two-year rat carcinogenicity study prior to knowing its outcome (ICH Status Report, 2016). Predictions in the submitted CADs will then be checked against the actual outcome of the two-year rat studies as they are completed. The results of this study are expected for 2019. Currently, the EPAA (European Partnership for Alternative Approaches to Animal Testing) is carrying out a project to evaluate whether a similar approach is also applicable to the carcinogenicity assessment of agrochemicals.

### 3.3 Reproductive and developmental toxicity

Reproductive toxicity is defined as “*effects such as reduced fertility, effects on gonads and disturbance of spermatogenesis; this also covers developmental toxicity*” (Ferrario et al., 2014), while developmental toxicity is defined as effects of “*e.g., growth and developmental retardation, malformations, and functional deficits in the offspring*”. Often referred to in combination as DART (developmental and reproductive toxicity), the assessment of these endpoints aims to identify possible hazards to the reproductive cycle, with an emphasis on embryotoxicity. Only 2–5% of birth defects are associated with chemical and physical stress (Mattison et al., 2010), including mainly the abuse of alcohol and other drugs, with a far greater percentage attributable to known genetic factors. Overall, approximately 50% of birth defects have unknown causes (Christianson et al., 2006). The available database is even more limited for the assessment of the prevalence of effects on mammalian fertility.

Similarly, DART was not in the foreground of updates to safety assessments for many years after the shock of the thalidomide disaster (Kim and Scialli, 2011) had died down. More recently, the European REACH legislation, which is extremely demanding in this field (Hartung and Rovida, 2009), has stirred discussion, notably because tests like the two-generation study are among the costliest and require up to 3,200 animals (traditional two-generation study) per substance. In the drug development area, the discussion has focused mainly around a possible replacement of the second species by human mechanistic assays and the value of using non-human primates for biologicals. Another driving force is the European ban on animal testing for cosmetics ingredients (Hartung, 2008b). A series of activities by ECVAM and CAAT, including several workshops, have tackled this challenge. The Integrated Project ReProTect (Hareng et al., 2005) was one of its offspring, pioneering several alternative approaches, followed by projects like Chem-Screen and most recently the flagship program EU-ToxRisk^4^ (Daneshian et al., 2016).

Developmental disruptions are especially difficult to assess (Knudsen et al., 2011), as the timing of processes creates windows of vulnerability, the process of development is especially sensitive to genetic errors and environmental disruptions, simple lesions can lead to complex phenotypes (and *vice versa*), and maternal effects can have an impact at all stages.

The treatment of one or more generations of rats or rabbits with a test chemical is the most common approach to identifying DART, detailed in seven OECD TGs. For specifically evaluating developmental toxicity, TGs were designed to detect malformations in the developing offspring, together with parameters such as growth alterations and prenatal mortality (Collins, 2006). For REACH, developmental toxicity tests are considered mainly as screening tests (Rovida et al., 2011). The shorter and less complex “screening” tests, which combine reproductive, developmental, and (optionally) repeated dose toxicity endpoints into a single study design, are variants.

The fundamental relevance of the current testing paradigm has only recently been addressed in a more comprehensive way (Carney et al., 2011; Basketter et al., 2012). There is considerable concern about inter-species differences (of about 60% concordance), reproducibility (in part due to a lack of standardization of protocols but also high background levels of developmental variants), and the value of the second generation in testing versus the costs, duration and animal use. An analysis of 254 chemicals (Martin et al., 2009b) suggests that 99.8% of chemicals show no-effect-levels for DART within a ten-fold range of maternal toxicity and thus might be simply covered by a safety assessment factor of 10.

An analysis by Bremer and Hartung (2004) of 74 industrial chemicals, which had been tested in developmental toxicity screening tests and reported in the EU New Chemicals Database, showed that 34 chemicals had demonstrated effects on the offspring, but only two chemicals were actually classified as developmentally toxic according to the standards applied by the national competent authorities (Bremer and Hartung, 2004).

This demonstrates the lack of confidence in the specificity of this “definitive” test. The same analysis showed that 55% of these chemical effects on the offspring could not be detected in multi-generation studies.

The status of alternative methods for DART has been summarized by Adler et al. (2011), endorsed by Hartung et al. (2011), and in the context of developing a roadmap to move forward by Basketter et al. (2012) and Leist et al. (2014). Some key developments are summarized in Table 3 and in the following text.

#### Extended one-generation reproductive toxicity study

Increasing doubt as to the usefulness of the second generation for testing of substances led to retrospective analyses by Janer et al. (2007), who concluded that this provided no relevant contribution to regulatory decision-making. The US EPA obtained similar data (Martin et al., 2009b) supporting the development of an extended one-generation study (OECD TG 443; OECD, 2011), originally proposed by the ILSI-HESI Agricultural Chemicals Safety Assessment (ACSA) initiative (Doe et al., 2006). The history of the new assay is summarized by Moore et al. (2009). This shows that elements of study protocols can indeed be useless and warrant critical assessment. The reduction brings the number of animals down from 3,200 to about 1,400 per substance tested. Ongoing discussions concern the new animal test’s modules for neurodevelopmental and developmental immunotoxicity, which may be triggered as a result of the extended one-generation study and which undo a lot of the reduction in terms of work and animal use.

#### Zebrafish embryotoxicity test

In the field of mammalian alternatives, the most complete reflection of embryonic development apparently can be achieved with zebrafish embryos (Selderslaghs et al., 2012; Sukardi et al., 2011; Weigt et al., 2010), for example using dynamic cell imaging, or frog eggs (FETAX assay) (Hoke and Ankley, 2005), with the latter having been evaluated quite critically by ICCVAM.

Currently, the Evidence-based Toxicology Collaboration (EBTC) is evaluating available protocols and data in a systematic review. This retrospective analysis is also exploring whether such systematic reviews (Stephens et al., 2016; Hoffmann et al., 2017) can substitute for traditional validation approaches (Hartung, 2010a). The US National Toxicology Program (NTP) is currently leading the Systematic Evaluation of Application of Zebrafish in Toxicity Testing (SEAZIT) project to assess the impact of varying protocol elements, harmonize ontologies, and develop recommendations around best practices.

#### Embryotoxicity tests

By 2002, three well-established alternative embryotoxicity tests had already been validated, i.e., the mouse embryonic stem cell test, the whole rat embryo culture and the limb bud assay (Genschow et al., 2004; Piersma et al., 2004; Spielmann et al., 2004). This decade-long validation process represented a radical departure from other validation studies ongoing at that time. They covered only a small, though critical, part of the reproductive cycle and embryonic development. For this reason, none of the tests have received regulatory acceptance in the 15 years since. Although the embryonic stem cell test was validated, the exact regulatory use was still to be defined (Spielmann et al., 2006). The validation study was criticized because the validity statements had raised significant expectations, but such partial replacements could only be used in a testing strategy (Hartung et al., 2013; Rovida et al., 2015) as later attempted within ReProTect and other projects cited above. This prompted a restructuring of the validation process with earlier involvement of regulators and their needs (Bottini et al., 2008), leading among other outcomes to today’s PARERE network at EURL ECVAM. This is only one example, but in general a common problem of tests that have undergone the classical validation process. This was also addressed and reflected on in the recently published ICCVAM strategic roadmap in conjunction with a clear statement “*The successful implementation of new approach methodologies (NAMs) will depend on research and development efforts developed cooperatively by industry partners and federal agencies. Currently, technologies too often emerge in search of a problem to solve. To increase the likelihood of NAMs being successfully developed and implemented, regulatory agencies and the regulated industries who will ultimately be using new technologies should engage early with test-method developers and stay engaged throughout the development of the technologies*.” (ICCVAM, 2018)

Other critical views as to the validation of alternative embryotoxicity tests concerned the low number of substances evaluated due to the costs of these assays, and the somewhat arbitrary distinction between weak and strong embryotoxicants, where a weak toxicant was defined as being reprotoxic in one species and a strong toxicant being reprotoxic in two or more.

Among the embryotoxicity tests, the murine embryonic stem cell test (EST) has attracted most interest, partly because it represents the only truly animal-free method of the three. Originally based on the counting of beating mouse embryonic stem cellderived cardiomyocytes, this test has been adapted to other endpoints and to human cells (Leist et al., 2008). It is also used in pharmaceutical industry with revised prediction models. A dedicated workshop on the problems of the EST (Marx-Stoelting et al., 2009) pointed out that its prediction model is overly driven by the cytotoxicity of compounds. Importantly, a variant of the EST using either human embryonic stem cells or human induced pluripotent stem cells and metabolite measurements, which were identified by metabolomics, was introduced by Stemina Biomarker Discovery. This CRO offers contract testing in-house. The assay was evaluated with very promising results for more than 100 substances and is undergoing evaluation by the US EPA and the NTP. There is ongoing discussion with the FDA whether such assays might replace the second species in DART evaluations.

#### Endocrine disruptor screening assays

Endocrine disruption is one key element of DART but may also constitute a pathway of carcinogenesis. The important assay developments in the context of chemical endocrine disruptor screening go beyond the scope of this short overview. However, they could form critical building blocks in an integrated testing strategy for DART as suggested first by Bremer et al. (2007) and attempted in ReProTect, and for carcinogenicity (Schwarzman et al., 2015).

#### Computational methods and the threshold of toxicological concern (TTC)

Development of (Q)SAR models for reproductive toxicity is relatively meagre, due to both the complexity of the endpoint and the limited available public data (Hartung and Hoffmann, 2009). The more recent availability of larger toxicity datasets might change this (Hartung, 2016).

An alternative approach has been made by improving TTC for DART (van Ravenzwaay et al., 2017) by expanding earlier attempts by BASF (Bernauer et al., 2008; van Ravenzwaay et al., 2011, 2012; Laufersweiler et al., 2012). The approach avoids testing by defining doses that are very unlikely to produce a hazard across a large number of chemicals based on the actual use scenario for a given substance of interest (Hartung, 2017c). This work resulted in remarkably high TTC (compared to other endpoints) of 100 μg/kg bw/day for rats and 95 μg/kg bw/day for rabbits for reproductive toxicity. If found acceptable, this could contribute to considerable test waiving.

## 4. Systems biology and toxicology

“*You think that because you understand ‘one’ that you must therefore understand ‘two’ because one and one make two. But you forget that you must also understand ‘and’*.” This quote by Donella H. Meadows in *Thinking in Systems: A Primer* hits the nail on the head: It is not about knowing the components but about their interrelationships. That is what systems approaches are about. The term has been used mainly for the computational approach of modelling these interrelationships. A key point made here is that there are two systems biology / toxicology approaches – one that is computational and one that is experimental – and they complement each other in addressing the complexity of the organism. Donella H. Meadows, quoted above, phrased it “*The behavior of a system cannot be known just by knowing the elements of which the system is made*”. We will ultimately propose to fuse these approaches, as we can sharpen our modeling tools with data generation in (quality-) controlled MPS. Mathematical modeling has a long history in physiology, but the new added value comes from the generation of big data via the respective measurement technologies (omics, high-content and sensor technologies), and the computational power to make sense of them.

### 4.1 Experimental systems biology and toxicology

We have recently comprehensively summarized the emergence of microphysiological systems (MPS) (Alépée et al., 2014; Hartung, 2014; Marx et al., 2016), which will not be repeated here. Here, the focus of this review will be on the understanding of how MPS can help to address systemic toxicities and aspects of their quality assurance. MPS bring a certain face-validity to the portfolio of tools as they introduce organ architecture, representative complexity and functionality to the *in vitro* approaches and increasingly even incorporate (patho-)physiological organ interactions. A critical element is the proper reflection of ADME, but microfluidics offers many opportunities to approach this goal (Slikker, 2014). The promise of MPS in biomedicine and drug development depends critically on their quality control. Especially, regulatory decisions based on them will require a high degree of confidence, which only strict quality control can create.

The quality assurance of MPS again requires an adaptation of the validation paradigm. Concepts of validation originally shaped around relatively simple cell systems for regulatory decision-taking as an alternative to animal testing. Three decades of experience have laid the foundation to broaden this concept to MPS in the context of their use in the life sciences and especially in drug development (Abaci and Shuler, 2015; Ewart et al., 2017; Skardal et al., 2016, 2017).

#### The FDA MPS program

FDA recognizes that alternative test platforms like organs-on-chip can give regulators new tools that are more predictive. However, for these new alternative methods to be acceptable for regulatory use, confidence is needed that the questions can be answered by these new methods as with traditional testing. Fostering collaborations between government researchers and regulators and between regulators, industry, stakeholders and academia can ensure that the most promising technologies are identified, developed, validated and integrated into regulatory risk assessment. The FDA-DARPA-NIH Microphysiological Systems Program started in 2011 to support the development of human microsystems, or organ “chips”, to screen swiftly and efficiently for safe and effective drugs (before human testing). It represents a collaboration through coordination of independent programs:

- Defense Advanced Research Projects Agency (DARPA): Engineering platforms and biological proof-of-concept (DARPA-BAA-11–73: Microphysiological Systems)
- National Institutes of Health (NIH), National Center for Advancing Translational Sciences (NCATS): Underlying biology/pathology and mechanistic understanding (RFA-RM-12–001 and RFA RM-11–022)
- Food and Drug Administration (FDA): Advice on regulatory requirements, validation and qualification.

**Fig. 2:**
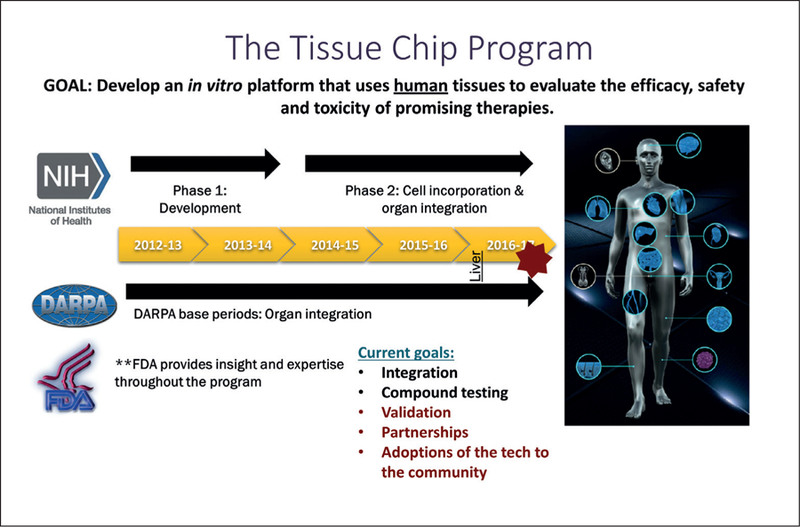
The FDA-DARPA-NIH Microphysiological Systems Program Abbreviations: NIH, National Institutes of Health USA; FDA, Food and Drug Administration USA; DARPA, Defense Advanced Research Projects Agency USA

This was a unique partnership because it involved regulatory scientists at the very beginning and thus was able to address identified gaps in knowledge needed to regulate FDA products (Fig. 2).

As an outcome of the program, in April 2017, the FDA signed a Cooperative Research and Development Agreement (CRADA) with Emulate, Inc. to use organs-on-chips technology as a toxicology testing platform to understand how products affect human health and safety. It aims to advance and qualify their “Human Emulation System” to meet regulatory evaluation criteria for product testing^5,6^. The FDA will evaluate the company’s “organs-on-chips” technology in laboratories at the agency’s Center for Food Safety and Applied Nutrition (CFSAN). Their miniature liver-on-chip will be evaluated as to its effectiveness to better understand the effects of medicines, disease-causing bacteria in foods, chemicals, and other potentially harmful materials on the human body. FDA will beta-test the Emulate system and look at concordance of chip data with *in vivo, in silico* and other *in vitro* (2-D) data on the same compounds; furthermore, FDA will begin to develop performance standards for organs-on-chips to create a resource for FDA regulators and researchers.

The work is part of the FDA Predictive Toxicology Roadmap announced December 6, 2017^7^. An FDA senior level toxicology working group was formed to foster enhanced communication among FDA product centers and researchers and leverage FDA resources to advance the integration of emerging predictive toxicology methods and new technologies into regulatory safety and risk assessments. This will include training of FDA regulators and researchers with continuing ongoing education in new predictive toxicology methods that are essential for FDA regulators. As part of this, FDA established an agency-wide education calendar of events and a Toxicology Seminar Series to introduce concepts of new toxicology methodologies and updates on toxicology-related topics. In order to promote continued communication, FDA reaffirmed its commitment to incorporate data from newly qualified toxicology methods into regulatory missions, is encouraging discussions with stakeholders as part of the regulatory submission process, and encourages sponsors to submit scientifically valid approaches for using a new method early in the regulatory process. FDA fosters collaborations with stakeholders across sectors and disciplines nationally and internationally. This is pivotal to identify the needs, maintain momentum, and establish a community to support delivery of new predictive toxicology methods. With this goal, FDA’s research programs will identify data gaps and support intramural and extramural research to ensure that the most promising technologies are identified, developed, validated, and integrated into the product pipeline. Under the oversight of the Office of the Commissioner, the progress of these recommendations will be tracked, including an annual report to the Chief Scientist. This shall ensure transparency, foster opportunities to share ideas and knowledge, showcase technologies, and highlight collaborations on developing and testing new methods.

In conclusion, the FDA roadmap identifies the critical priority activities for energizing new or enhanced FDA engagement in transforming the development, qualification, and integration of new toxicology methodologies and technologies into regulatory application. Implementation of the roadmap and engagement with diverse stakeholders should enable FDA to fulfill its regulatory mission today while preparing for the challenges of tomorrow.

#### Validation of M(P)PS

Quality assurance and ultimately validation of the tools in the life sciences is a key contribution to overcome the stagnant drug development pipeline due to high attrition rates and the reproducibility crisis in biomedicine. MPS bring a certain face-validity to the portfolio of tools as they introduce organ architecture and functionality to the *in vitro* approaches and increasingly even incorporate (patho-)physiological organ system interactions. With more MPS developing, the major challenge for their use as translational drug development tools is to make micropathophysiological systems (MPPS). The promise of MPS in biomedicine and drug development depends critically on their quality control. Especially, regulatory decisions will require a confidence that only strict quality control can create.

Typically, the new test would be compared to a traditional method, usually an animal experiment, and the relative reproducibility of reference results would be used as the primary measure of success. Concurrent testing of new substances with the reference test represents another opportunity to gain comparative information without the information bias of the scientific literature (e.g., overrepresentation of toxic substances with specific mechanisms). In an ECVAM workshop (Hoffmann et al., 2008), it was suggested that instead of a specified reference (animal) test, a reference standard could be formed by expert consensus by integrating all knowledge; a list of substances could be produced with results a hypothetical ideal test would provide. This can for example allow using also human data in combination with animal data or combination of results from various test systems.

These concepts of correlative validation are only partially applicable to MPS, which often have many purposes other than replacing an animal test, and for which in many cases a respective animal test does not even exist. For drug development, typically a pathophysiological state first needs to be introduced and then treatment effects are analyzed. This greatly complicates the validation process, as both the induction of pathophysiology and its correction need to be quality assured.

MPS are usually more relevant based on the mechanisms of physiology and pathophysiology they reflect. For this reason, mechanistic validation (Hartung et al., 2013) lends itself to the evaluation of MPS: This is first of all a comparison to mechanisms from the scientific literature, ideally by systematic review. Alternatively, high-content characterizations of a reference test and the new test can show that similar patterns of perturbation of physiology occur, in the easiest case that the same biomarkers of effect are observed. This experimental approach can be applied where the definition of mechanism is incomplete or the existing literature insufficient. Lastly, computational modelling of physiology and the prediction of test outcomes in comparison to real test data can show how well the test and the computational model align. The envisaged validation process for MPS has to start with the information need, which defines the purpose of the test.

**Fig. 3:**
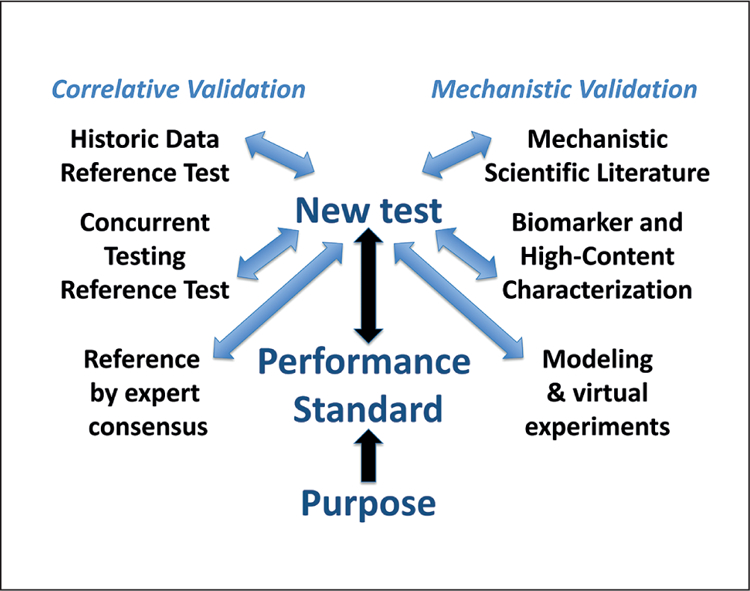
The concept of performance standard-based validation The different elements for anchoring a validation in a correlative or mechanistic manner will be combined by expert consensus to define a performance standard meeting a test purpose.

Although validation is often perceived as rigid and inflexible (which it has to be once a study is initiated), it is actually a highly flexible process, which needs to be adapted case by case and should be performed with the end use in mind (ICCVAM, 2018). Concepts of pre-validation, applicability domain, retrospective validation, catch-up-validation, minimal performance standards, prediction models, etc. are examples of the continuing adaptation to meet the needs of stakeholders (Hartung, 2007; Leist et al., 2012). Here, especially the concepts of “fit-for-purpose”, meeting defined “performance standards”, and “mechanistic validation” will have to be elaborated upon, specific to MPS (Fig. 3).

- “Fit-for-purpose”: The purpose of a test is its place and function in a testing strategy to meet an overall information need and decision context, e.g., the information need is developmental neurotoxicity with the focus on one of the key events of neural development such as myelination of axons. The question to be addressed can be the following: Do certain substances perturb myelination of neuronal axons? Then, a first test could assess toxicity to oligodendrocytes. A second test could quantify the level of myelin basic protein (MBP) in MPS. A third test might assess electrophysiology within the organoid as a functional outcome of perturbed myelination and as a consequence of the perturbation of neurodevelopment and neural differentiation. The testing strategy would need to combine these test results (evidence integration) with existing information.
- “Performance standards”: The concept of a performance standard for alternative methods was introduced in the Modular Approach in 2004 (Hartung et al., 2004) and incorporated into OECD validation guidance from 2005 (OECD, 2005). The basic idea is that if a successfully validated method is available, it should be defined what a similar method should demonstrate to be considered equivalent to the validated one. This has proven to be crucial for any modification of tests as well as to avoid extensive and expensive retesting for similar tests. For this reason, they were originally termed “minimum performance standards”. Over time, the concept has evolved, now also using the performance standards among others to show the proficiency of a laboratory to carry out a test. Most radically, the current work on developing a performance standard-based OECD TG for a skin sensitization defined approach (DA) aims at defining how any test or combination of tests should perform to be acceptable under the guidance without prescribing a specific method. By extension, a performance standard could be defined for an MPS: This means setting engineering goals (performance standards) and the quality assurance (validation) process would confirm that these standards are met. To some extent this is similar to the reasoning of an earlier ECVAM workshop on points of reference, where it was recommended to define a point of reference by expert consensus for a given validation, not by comparing to a dataset from a traditional animal test (Hoffmann et al., 2008). This was first applied in the retrospective validation of the micronucleus *in vitro* test validation (Corvi et al., 2008) and later in the more recent validation studies of micronucleus and comet assays in 3D skin models, and it will be applied in the future for the validation of thyroid endocrine disruptor tests.
- “Mechanistic validation” (Hartung et al., 2013) is another radical departure from current practice. Though validation has always included the aspect of mechanistic relevance when addressing test definition, this is usually only minimally covered. The traditional (animal) test and the new method are typically taken as black boxes and the correlation of their results is the measure of validity. MPS bring (patho)physiology, i.e., mechanism, to the foreground. Thus, it makes sense to use a mechanistic basis for comparison. Mechanistic validation dictates first an agreement on the relevant mechanisms for a given information need, followed by evaluation based on coverage of the mechanism by the new method. This type of an approach increasingly takes place with the definition of adverse outcome pathways (AOP) and was the goal of the parallel Human Toxome Project (Bouhifd et al., 2015). One of the basic underpinnings of mechanistic validation is that a systematic review of the literature can be used to ascertain mechanism.

Even before attempting formal validation of MPS, their quality assurance will be of utmost importance. The Good Cell Culture Practice (GCCP) movement initiated by one of the authors in 1996 led to the first guidance of its kind (Coecke et al., 2005) under the auspices of ECVAM. The international community recognized a need to expand this to MPS and stem cell-based models ten years later, and under the lead of CAAT, with participation of FDA, NIH NCATS, NICEATM, ECVAM, UK Stem Cell Bank and others, GCCP 2.0 was initiated. In two dedicated workshops and several publications (Pamies et al., 2017b, 2018b; Pamies and Hartung, 2017; Eskes et al., 2017), the needs were defined, and a steering group plus scientific advisory group is currently formulating GCCP 2.0. The proof-of-principle of validation attempts by NIH NCATS in establishing Tissue Chip Testing Centers (TCTC) will cross-fertilize with these developments. The GCCP discussion was already the topic of workshops and conferences such as European Society of Toxicology *In Vitro* 2016, EuroTox 2017, Society of Toxicology 2018 and a joint conference with FDA and the IQ consortium in 2015. A 2017 workshop (Bal-Price et al., 2018) developed test readiness criteria for toxicology using the example of developmental neurotoxicity, which will be a further starting point for the performance standard development attempted here.

Recognizing the need for a stakeholder dialogue on the quality assurance of MPS, CAAT this year initiated a Public Private Partnership for Performance Standards for Microphysiological Systems (P4M), which aims to establish a stakeholder consensus process toward performance standards. P4M will discuss the core aspects, i.e., when is an MPS good enough and can we express this as a performance standard? Expressions of interest already received include various companies, academics, ECVAM, and US and Japanese agencies.

### 4.2 Computational systems biology and toxicology

J. B. S. Haldane (1892–1964), a biologist and mathematician, predicted “*If physics and biology one day meet, and one of the two is swallowed up, that one will be biology*”. Systems biology is biology swallowed by physics. Joyner and Pederson (2011) give an interesting reflection on this discipline. Systems toxicology (Kiani et al., 2016), its more applied sibling, was the topic of an earlier article in this series (Hartung et al., 2012), some dedicated conferences and symposia (Andersen et al., 2014; Sturla et al., 2014; Sauer et al., 2015; Hartung et al., 2017a) and a special issue of *Chemical Research in Toxicology* (Hartung et al., 2017b). As experimental systems biology has been fueled by bioengineering and stem cell technologies, computational systems biology / toxicology has been driven by big data and machine learning technologies (Hartung, 2016; Luechtefeld and Hartung, 2017). The ultimate vision is using computational models of human metabolism, possibly as avatars or virtual patients, to try out pharmacological interventions or toxic insults; on the way, tissue and organ models are emerging (Hartung, 2017d).

Systems biology approaches biological function and its perturbation by various biochemically active compounds by complementing the traditional reductionist approaches. The emphasis of systems biology approaches is on the interactions between components rather than just the components themselves. This approach is therefore frequently focused on the dynamics of biological interactions and the emergent properties of biological cells, tissues and organisms stemming from the complexity of the underlying regulatory networks.

The systems biology analysis allows one to examine the disruption of network components by pharmacological and other interventions through the lens of their effects, not only on the designated target but on the network of molecular components, with frequently paradoxical, unexpected and counter-intuitive results. These results can be products of complex feedback interactions involving a specific target and the multiple phenotypes controlled by it, rather than just off-target biochemical effects. The network level effects can span multiple scales, from biochemical to cellular and tissue levels, which involve cell-cell communication through various signaling mechanisms, producing networks of networks. This complexity is captured through high-throughput experimentation and computational analysis and modelling, with a particular focus on the unanticipated, emergent properties. Below we provide some examples of the recent systems biology approaches to complex problems related to the mechanisms of drug action and possible toxic effects.

Several recent examples of the systems approach illustrate the philosophy and power of the approach. Particular attention so far has been paid to the complex mechanisms of action of cocktails of various pharmaceutical compounds. For instance, a recent systems analysis demonstrated that the order and timing of application of anti-cancer compounds can determine the efficacy of combinatorial treatments (Lee et al., 2012). This effect has been ascribed to re-wiring of the signaling network by the first compound, which might result in a more potent effect of the second compound if applied at the appropriate time. The same dynamic network view can be applied to combinatorial applications of radio- and chemotherapeutic treatments, as elucidated through mathematical modeling and experimental validation in another high-profile systems biology application (Chen et al., 2016). These types of network perspectives and associated modeling support will likely also inform the analysis of the effects of putative combinatorial treatments on other tissues and the associated toxicology outcomes.

Another example of a study benefiting from a systems approach is the paradoxical increase rather than decrease of the total kinase activity by ATP-competitive inhibitors of BRAF/ CRAF kinases (Hatzivassiliou et al., 2010). As these kinases are a key target in various cancers, the paradoxical effect has received much attention. However, it is virtually impossible to rationalize it without a modeling approach within a framework of systems biology. Two key insights had to be made to formulate hypotheses of how this might occur, including a feedback regulation inherent in the RAF/MAPK kinase signaling and potential allosteric action of the drugs on the enzyme (Kholodenko et al., 2015).

A number of recent efforts to build and apply computational systems models have focused specifically on mechanisms of developmental toxicity. The US EPA’s Virtual Tissues research project uses cellular agent-based models to recapitulate developing embryonic systems and creates *in silico* testing platforms by parameterizing such models using the ToxCast/Tox21 HTS data to mimic chemical exposure and simulate effects on a tissue level. An AOP of embryonic vascular disruption was published based on a systematic literature review (Knudsen and Kleinstreuer, 2012) and was used to inform the construction of a computational model predicting disruption of blood vessel development (Kleinstreuer et al., 2013b). Putative vascular disruptor compounds and associated systems model predictions have been tested and confirmed in a number of functional assays such as transgenic zebrafish, human cell-based tubulogenesis assays, and whole embryo culture (Tal et al., 2016; McCollum et al., 2016; Ellis-Hutchings et al., 2017). Other work has focused on modelling key developmental toxicity mechanisms driving cleft palate formation (Hutson et al., 2017) and taking a systems toxicology approach to understanding disruption of male reproductive development and endocrine pathways (Leung et al., 2016; Kleinstreuer et al., 2016)

### 4.3 A fusion of experimental and computational systems biology / toxicology?

Even though MPS are complex, they are considerably simpler than human organisms and they are much more open for measurements and interventions. Thus, the opportunity to first model our experimental systems has enormous advantages; however, it represents an interdisciplinary challenge. Bioengineers and modelers have to be brought together. At the same time, funding bodies have to be convinced of the value of this interim step. As an example, in a recent organ-on-chip study (Kilic et al., 2016), mediator gradients were modeled computationally and and then verified experimentally. By parameterization of the experimental systems, we can also start to scale our systems virtually as a quantitative *in vitro* to *in vivo* extrapolation (QIVIVE) (Tsaioun et al., 2016; Hartung, 2018).

## 5 Conclusions

Overall, further discussion is needed as to the relevance of current carcinogenicity, RDT and DART assessments. Recognizing the societal need to ensure the safety of drugs, chemicals and consumer products, this might make it difficult to abandon current testing, but should lower the barrier for implementing alternative approaches that may improve the status quo. Data sharing and the harmonization of ontologies and data formats will be critical.

Repeated-dose toxicity, carcinogenicity and reproductive toxicity are three examples of systemic toxicology implementation. Since they are very complex endpoints, the uptake of alternative *in vitro* test methods is still very limited. Rather, some approaches are being investigated or are already in place for waiving testing and reducing the number of animals, such as the ICH strategy for pharmaceuticals and the extended one-generation assay.

Promise for all systemic toxicities comes from the starting development of integrated testing strategies driven by mechanistic relevance: By mapping the human reproductive cycle and its disturbance or the array of pathways of carcinogenesis with a number of assays, the hope is to design more human-relevant test strategies. These and other approaches form part of the emerging roadmap for replacement (Basketter et al., 2012; Leist et al., 2014; Corvi et al., 2017; ICCVAM, 2018) and will contribute to the momentum for implementing alternative approaches, which is also aided by the increasing recognition of the shortcomings of current testing methods.

Given the importance of these hazards and the backlog of testing for most substances of daily use, more efforts in the development of tests, design of testing strategies and their validation are needed. Quality assurance and ultimately validation of the tools in the life sciences is a necessity to unplug the drug development pipeline, which is blocked by high attrition rates, and the reproducibility crisis in biomedicine. The systematic condensation of our existing knowledge (including the mapping of gaps and shortcomings of existing evidence) can herald a more predictive systems approach to addressing systemic toxicity.

However, ultimately, we need a “new deal” for systemic toxicities. Albert Einstein once said, “*We can’t solve the problems by using the same kind of thinking we used when we created them*”. The increasing awareness of the shortcomings of current tests with respect to reproducibility (Baker, 2016; Jilka, 2016; Voelkl et al., 2018), inter-species differences and thus lack of human relevance, ambiguity of results and steep costs, should make all who are in this field uneasy (Miller, 2014). It requires the “art of toxicology” to make good decisions on the basis of such compromised information sources. How can we sleep well when we know that our daily decisions are subject to these limitations? Rasheed Ogunlaru wrote, “*All the tools, techniques and technology in the world are nothing without the head, heart and hands to use them wisely, kindly and mindfully*”. This holds for the current art of toxicology and will likely be no different for any new approach. Especially as the new approaches come in the guise of objective “evidence-based” and high-tech approaches, they are still models created with a purpose. Frank Herbert (in God Emperor of Dune) warns “*Dangers lurk in all systems. Systems incorporate the unexamined beliefs of their creators. Adopt a system, accept its beliefs, and you help strengthen the resistance to change*”.

The 3Rs have served us to some extent to replace animal tests for acute and topical toxicities but have done so by modelling and reproducing the animal test results despite their shortcomings. A testing strategy modelling the outcomes of traditional tests will not serve us as well for systemic toxicities. The hazards are typically more severe and less directly attributable to exposure because they can manifest anywhere in the body and after any time of exposure. The 3S approach suggested here is such a “new deal” for safety assessments. It goes far beyond the 3Rs as it does not aim to reproduce the results of a black box (animal) test, which may bear little resemblance to the human scenario. The combination of systematic evaluation of our knowledge and experimental as well as computational modelling of biological systems complexity promises a different approach to systemic toxicity prediction, even though it still has to prove its feasibility and utility.

## Figures and Tables

**Tab. 1:** Worrisome analyses as to the relevance of traditional systemic toxicity studies

Repeated-Dose Toxicity (RDT)	Developmental and reproductive toxicity (DART)	Cancer bioassay
Interspecies concordance of mice with rats (37 chemicals): 57%–89% (average 75%) in short-term and 65%–89% (average 80%) in long-term studies (Wang and Gray, 2015)	No relevant contribution to regulatory decision-making by second generation testing (Janer et al., 2007; Martin et al., 2009a)	While 53% of all chemicals test positive, age-adjusted cancer rates did not increase over the last century (Jemal et al., 2009)
Mouse-to-rat organ prediction (37 chemicals) in long-term studies with an average of 55%, in short-term studies with an average of 45%. For rat-to-mouse, the averages were 27% and 49%, respectively (Wang and Gray, 2015)	254 chemicals in ToxRefDB tested in both multi-generation and 2-year chronic studies, and 207 chemicals tested in both multigeneration and 90-day subchronic studies (Martin et al., 2009b); with an assessment factor of 10, the hazard of reproductive toxicity might be covered for 99.8 % of substances	Exposure to mutagens does not correlate with oncomutations in people (Thilly, 2003)
Species concordance (310 chemicals) for non-neoplastic pathology between mouse and rat was 68% (Wang and Gray, 2015)	No experience for industrial chemicals: < 25 two-generation-studies and < 100 one-generation studies in EU and US in 30 years (Bremer et al., 2007)	Protocol has poorly defined endpoints and a high level of uncontrolled variation; could be optimized to include proper randomization, blinding, better necroscopy work, and adequate statistics (Freedman and Zeisel, 1988).
Inter-species differences mouse vs. rat (95^th^ percentile) of 8.3 for RDT (Bokkers and Slob, 2007)	Large number of individual skeletal variations (sometimes > 80%) even in control animals (Daston and Seed, 2007)	Most recent test guidelines (OECD, 2009) still do not make randomization and blinding mandatory, and statistics do not control for multiple testing, although about 60 endpoints are assessed (Basketter et al., 2012).
Low correlation between 28-day and 90-day NOAEL for 773 chemicals (Luechtefeld et al., 2016b, Fig.4)	Of those agents thought not to be teratogenic in man, only 28% are negative in all species tested (Brown and Fabro, 1983)	Not standardized for animal strains (“*young healthy adult animals of commonly used laboratory strains should be employed*”) ( Basketter et al., 2012)
A limited set of only six targets consisting of liver, kidney, clinical chemistry, body weight, clinical symptoms and hematology within a study gives a probability of 86% to detect the LOEL (Batke et al., 2013)	Of 1223 definite, probable and possible animal teratogens, fewer than 2.3% were linked to human birth defects (Bailey et al., 2005)	Problems with standardization of strains that hamper the use of historical control groups (Haseman et al., 1997): the most commonly used strains showed large weight gain and changes in some tumor incidences that resulted in reduced survival over just one decade (attributed to intentional or inadvertent selection of breeding stocks with faster growth and easier reproduction)
	Not robust with about 25% equivocal studies (Bailey et al., 2005)	Analysis of 1,872 individual species/gender group tests in the US National Toxicology Program (NTP) showed that 243 of these tests resulted in “equivocal evidence” or were judged as “inadequate studies” ( Seidle, 2006)
	74 industrial chemicals tested in New Chemicals Database: 34 showed effects on offspring, but only 2 chemicals were classified as developmental toxicants (Bremer and Hartung, 2004)	Questionable two-species paradigm as rats are more sensitive, and regulatory action is rarely taken on the basis of results in mice (Van Oosterhout et al., 1997; van Ravenzwaay, 2010)
	55% of positives in screening studies not in multi-generation studies (Bremer and Hartung, 2004)	Concordance of 57% comparing 121 replicate rodent carcinogenicity assays ( Gottmann et al., 2001)
	Group size limits statistical power (Hotchkiss, 2008)	The apparent correlation between potency of carcinogens in mice and rats is largely an artifact (Bernstein et al., 1985).
	61% inter-species correlation (Hurtt, 2003; Bailey et al., 2005)	Concordance of 57% between mouse and rat bioassays (Gray et al., 1995).
	Given 2.5% true reproductive toxicants and 60% inter-species correlation, testing with two species will find 84% of the toxic but label 64% of the negatives falsely (Hartung, 2009b)	Less than 50% probability for known carcinogens that induce tumors in one species in a certain organ to also induce tumors in another species the same organ comparing rats, mice, and hamsters, as well as humans (Gold et al., 1991, 1998).
	Of 38 human teratogens, the following percentages tested positive in other species: mouse 85%, rat 80%, rabbit 60%, hamster 45%, monkey 30%, two or more species 80%, any one species 97% (Brown and Fabro, 1983)	Doses are hundreds to thousands of times higher than normal exposures and might be carcinogenic simply because they overwhelm detoxification pathways (Schmidt, 2002)
	Of 165 human non-teratogens, the following percentages tested negative in other species: mouse 35%, rat 50%, rabbit 70%, hamster 35%, monkey 80%, two or more species 50%, all species 28% (Brown and Fabro, 1983)	69% predictivity of human carcinogenicity for the two-species cancer bioassay (Pritchard et al., 2003)
	Reproductive toxicity within 10-fold of maternal repeated-dose toxicity for 99.8% of 461 chemicals (Martin et al., 2009b)	In 58% of cases considered by the EPA, the positive cancer bioassay was insufficient for assigning human carcinogenicity ( Knight et al., 2006a,b )
		Cancer bioassays in nonhuman primates on 37 compounds were “… *inconclusive in many cases*” but carcinogenicity was shown unequivocally for four of them ( Takayama et al., 2008)
		About 50% of all chemicals tested positive in the cancer bioassay test (Basketter et al., 2012), and 53% of 301 chemicals tested by the NTP were positive, with 40% of these positives classified as non-genotoxic (Ashby and Tennant, 1991)
		An early analysis of 20 putative human non-carcinogens found 19 false-positives, suggesting only 5% specificity (Ennever et al., 1987).
		Only one in ten positive compounds is truly carcinogenic (Rall, 2000)
		Not all human carcinogens are found: Diphenyl-hydantoin (phenytoin) (Anisimov et al., 2005); the combination of aspirin/ phenacetin/ caffeine (Ennever and Lave, 2003); asbestos, nickel, benzidine-like compounds (Johnson, 2001); no cigarette smoke-induced lung cancer, no rodent leukemia induced by benzene, and no genetic point mutations induced by arsenic (Silbergeld, 2004).
		Estimate 70% sensitivity as well as specificity, assuming 10% real human carcinogens (Lave et al., 1988)
		Of 167 chemicals that caused neoplastic lesions in rat or mouse chronic/cancer studies, 35% caused neoplastic lesions in both rat and mouse (Martin et al., 2009a)
		Increasing the number of animals per group from 50 to 200 would result in statistically significant (p < 0.01) dose-responses for 92% of substances tested (Gaylor, 2005)

**Tab. 2:** Milestones on the road towards a new approach to carcinogenicity testing

Date	Event	Who was involved
1995	Joint proposal for a new OECD TG for the *in vitro* Syrian Hamster Embryo (SHE) Cell Transformation Assay (CTA)	USA and France
1998	Workshop on “Cell transformation assays as predictors of carcinogenicity”	ECVAM
2006	Workshop on “How to reduce false positive results when undertaking *in vitro* genotoxicity testing and thus avoid unnecessary follow-up animal tests”	EURL ECVAM
2006–2011	EU-6 Framework Project CarcinoGENOMICS	DG RTD / EU Consortium
2011	ESAC Opinion on prevalidation of *in vitro* Syrian Hamster Embryo (SHE) Cell Transformation Assay	EURL ECVAM
2012	ESAC opinion on validation of *in vitro* Bhas42 Cell Transformation Assay	JaCVAM /EURL ECVAM
2009	Acceptance of transgenic models as alternative to bioassay in second species	ICH
2013	ICH Regulatory Notice Document announcing the evaluation of an alternative approach to the 2-year rat carcinogenicity test	ICH and Drug Regulatory Authorities
2015	Starting activity on IATA for non-genotoxic carcinogens	OECD
2015	Adoption of Guidance Document No. 214 on the *in vitro* Syrian Hamster Embryo (SHE) Cell Transformation Assay	OECD
2016	Adoption of Guidance Document No. 231 on the *in vitro* Bhas42 Cell Transformation Assay	OECD
2016	Inclusion of characteristics of carcinogens in systematic reviews for Monograph program	IARC
2017	Initiation of the project on predicting carcinogenicity of agrochemicals	EPAA

Abbreviations: DG RTD, EU Directorate General Research and Technological Development; ECVAM, European Centre for the Validation of Alternative Methods; EPAA, European Partnership for Alternative Approaches to Animal Testing; EURL, European Reference Laboratory; IARC, International Agency for Research on Cancer; ICH, International Council for Harmonisation of Technical Requirements for Pharmaceuticals for Human Use; JaCVAM, Japanese Center for the Validation of Alternative Methods; OECD, Organisation for Economic Co-operation and Development

**Tab. 3:** Milestones on the road towards a new approach to DART

Date	Event	Who was involved
2002	Validation of three embryotoxicity tests	ECVAM, ZEBET, RIVM
unclear	Zebrafish for DART	Many groups, currently validated by EBTC
2005–2010	ReProTect	ECVAM, University Tübingen (Coordinator Michael Schwarz), 35 partners
2009	Stemina DevTox assay commercially available	Stemina
2012	Acceptance of extended one-generation reproductive toxicity study	ECVAM, ECHA
2008–2017	Definition of TTC	BASF SE, CAAT
2017	Draft Guidance “Detection of toxicity to reproduction for human pharmaceuticals” including suggested reference chemicals for characterizing alternative DART assays	ICH

Abbreviations: BASF SE, German chemical company; CAAT, Center for Alternatives to Animal Testing at Johns Hopkins University; EBTC, Evidence-based Toxicology Collaboration; ECHA, European Chemicals Agency; ECVAM, European Centre for the Validation of Alternative Methods; ICH, International Council for Harmonisation of Technical Requirements for Pharmaceuticals for Human Use; RIVM, Netherlands National Institute for Public Health and the Environment; ZEBET – Center for Documentation and Evaluation of Alternative Methods to Animal Experiments at the German Federal Institute for Risk Assessment

## Footnotes

1 http://www.ebtox.org

2 https://bit.ly/2q8yXXh

3 http://www.dcn.ed.ac.uk/camarades/#carousel-example-generic

4 http://www.eu-toxrisk.eu

5 https://emulatebio.com/press/fda-collab-agreement-emulate/

6 https://bit.ly/2ovDVNC

7 https://bit.ly/2ixBTID
